# Influence of Soluble Fiber as a Carrier on Antioxidant and Physical Properties of Powders Produced Based on the Spray Drying of *Malvae arboreae flos* Aqueous Extracts

**DOI:** 10.3390/foods12183363

**Published:** 2023-09-07

**Authors:** Katarzyna Lisiecka, Dariusz Dziki, Urszula Gawlik-Dziki, Michał Świeca, Renata Różyło

**Affiliations:** 1 Department of Biochemistry and Food Chemistry, University of Life Sciences in Lublin, Skromna St. 8, 20-704 Lublin, Polandurszula.gawlik@up.lublin.pl (U.G.-D.);; 2 Department of Thermal Technology and Food Process Engineering, University of Life Sciences in Lublin, Głęboka St. 31, 20-612 Lublin, Poland; 3 Department of Food Engineering and Machines, University of Life Sciences in Lublin, Głęboka St. 28, 20-612 Lublin, Poland

**Keywords:** black hollyhock flowers, spray drying, inulin, pectin, flavonoids, anthocyanins

## Abstract

The objective of this study was to assess the impact of inulin and pectin, wherein pectin replaced inulin with weight ranging from 2% to 8%, as wall materials on various aspects: bioactive component content, antioxidant and anti-inflammatory properties, bioavailability, powder recovery during the drying process, and selected physical characteristics of powders derived from *Malvae arboreae flos* aqueous extracts obtained through spray drying. Powders containing a soluble fraction of fiber demonstrated a recovery efficiency of over 50% during drying, along with low moisture content, water activity, and hygroscopicity, coupled with high solubility. The incorporation of pectin up to 8% did not significantly alter the color profile of the powders. However, at levels of 4% to 8% pectin, concave distortions and particle morphology cracks became noticeable, along with the potential to form agglomerates (evident when the span index ranged between 5.11 and 14.51). The substitution of inulin with pectin led to higher total contents of flavonoids (from 1.31% to 49.57% before digestion, and from 18.92% to 36.48% after digestion) and anthocyanins (from 45.79% to 78.56% before digestion, and from 65.45% to 521.81% after digestion) compared to samples containing only inulin as a carrier. Bioacceptability values exceeding 100% indicated effective preservation of compounds responsible for ferric-reducing antioxidant power, as well as the inhibition of xanthine oxidase and cyclooxygenase-2 across all samples.

## 1. Introduction

*Malvae arboreae flos* (MAF) is also known as *Althaea rosea (L.) Cav. var. nigra* [1] or black hollyhock flowers [2]. MAF can be found in gardens in Southern Europe, the Middle, Near East, the Mediterranean, the Central Asian region, and China. Plants from the *Malvaceae* family are commonly used in tea therapy, especially *Althaea rosea* and *Althaea officinalis* [3]. In folk medicine, the hollyhock flowers are primarily appreciated for ailments of the respiratory, urinary, and digestive tracts, as they possess anti-inflammatory properties and are applied externally in the case of ulcers [1). The plant’s medicinal value is attributed to the fact that it is a source of anthocyanins and flavonoids, including quercetin and kaempferol [4]. Additionally, it has been observed that the flowers of this plant are rich in polyphenols, showing antioxidant activity [5]. Several phenolic acids have been identified in the methanol–water extract of hollyhock flowers: *p*-coumaric (mainly), caffeic, *p*-hydroxybenzoic, and ferulic acids [1]. Therefore, MAF may serve as an additional source of health-promoting ingredients in new food products. This is especially relevant since the consumption of infusions is influenced by various factors and the lack of cultural or health habits or sensory intolerance can affect our choice [6].

The use of an MAF extract and carriers, such as inulin or pectin during spray drying, may bring additional nutritional properties to the final powder, especially since both polysaccharides are soluble fiber fractions [7,8]. Inulin is mainly produced from chicory and belongs to a group of indigestible carbohydrates called fructans. It has been proven to promote the development of microflora, and is considered an appropriate dietary component for people suffering from diabetes, as it does not increase blood glucose levels [9]. Pectins are commonly found in plant cell walls. In chemical terms, they consist of chains of galacturonic acid interspersed with rhamnose units and branched with chains of pentose and hexose units. They are credited with the ability to lower cholesterol, which results from their gelling properties [10]. Technologically, soluble fiber fractions are used, e.g., as emulsifiers, fillers, stabilizers, or thickeners, and they affect the final texture of the product. They are often used as fat or sugar substitutes, reducing the product’s energy value [11]. Inulin and pectin have also been used as carriers for spray drying [11,12,13]. However, it should be noted that despite the potential advantages of using polysaccharides as carrier materials, there are also limitations, particularly in terms of their solubility. Therefore, in many studies, it can be found that carriers consist of at least two polysaccharides, in order to eliminate the disadvantages resulting from their natural properties [14]. Additionally, it is considered appropriate to measure bioaccessibility when assessing the behavior of selected carriers in relation to active substances. This provides information on the proportion of a compound consumed in a meal that is released from the food matrix during digestion and absorption in the small intestine [15].

The aim of this study was to investigate the effect of the carrier combination, i.e., inulin and pectin, on the selected physical properties and the content of active compounds, as well as potential antioxidant and anti-inflammatory activity before and after digestion of powders obtained as a result of spray drying of water extracts based on MAF with a soluble fraction of fiber.

## 2. Materials and Methods

### 2.1. Basic Materials

Dehydrated MAF were obtained from AGREST Sp. z o.o. (Lublin, Poland) and are characterized in Table 1. Chicory inulin (Agnex, Białystok, Poland) and amidated pectin (C&G Sp. z o.o., Jastków, Poland) were used as carrier agents.

### 2.2. Feed Solution Preparation and Spray Drying Process

Initially, an infusion of *Malva arborea floss* was prepared in a ratio of 1:20 with water at a temperature of 95 °C. The mixture was then allowed to cool, strained, and centrifuged at 5000× *g* rpm using an MPW-260R centrifuge (MPW, Warsaw, Poland). The supernatant was lyophilized at −40 °C. The lyophilisate was stored in plastic Ziplock bags at 20 °C. 

Solutions of coating materials were prepared by dissolving inulin (I) in an amount from 23 g to 25 g in 200 mL of distilled water and adding pectin (P) from 0 g to 2 g to obtain 12.5% (*w*/*v*) of solids in all 5 solutions (M1—100% inulin in wall material, M2—98% inulin in wall material and 2% pectin in wall material, M3—96% inulin in wall material and 4% pectin in wall material, M4—94% inulin in wall material and 6% pectin in wall material, and M5—92% inulin in wall material and 8% pectin in wall material). Next, the blends were mixed and heated using a 06-MS-S280PRO hot magnetic stirrer (Chemlad, Stargard, Poland) to obtain a homogenous solution and then cooled down to the ambient temperature. The final feed solution was mixed with a 1% solution of a water extract solution of lyophilisate MAF and 12.5% solutions of coating materials in a ratio of 1:5 (*v*/*v*). Before spray drying, blends were homogenized using a hand blender, MBL-03 (MPW, Milanówek, Poland), for 10 s. 

The spray drying process was carried out using a Mini Spray Dryer Büchi B-290 (Büchi Labortechnik AG, Flawil, Switzerland). The inlet temperature of the drying air was 140 °C and the outlet temperature was 50 °C. The flow rate of the feed solution was set at 8 mL/min. The flow rate of compressed air supplied to the spray nozzle (about a 0.7 mm internal diameter) was 0.742 m^3^/h, and the drying air was 38 m^3^/h. The process parameters were chosen empirically. The collected powders were weighed, packed in plastic bags, and stored at −20 °C. The resulting microcapsules were tested on the following day after drying.

### 2.3. Properties of the Drying Process and Powders

#### 2.3.1. Process Yield

The process yield (YE) was calculated from the following formula proposed by Castro-Munoz et al. [16]:

(1) $$YE=\frac{w_{1}}{w_{2}}*100$$

where YE is the process yield (%), w_1_ is the mass of powder collected after the drying process (g), and w_2_ is the mass of solids dissolved in the feed solution (g).

#### 2.3.2. Moisture Content and Water Activity

Moisture measurement was determined using a Sartorius MA35 moisture analyzer (Göttingen, Germany), and water activity in the tested material was measured using LabMaster-aw (Novasina AG, Switzerland). In both cases, 0.1 g of the test material was weighed, and an automatic measurement was made. The audible signal informed the end of treatment.

#### 2.3.3. Hygroscopicity

The climate chamber MLR-352H-PE phcbi (Etten-Leur, the Netherlands) was used for the measurement of hygroscopicity. One gram of powder was placed in containers and on the chamber’s middle shelf. The temperature inside the device was 25 °C and the relative humidity was 75%. The study took place for 1 week (containers with powders were then weighed after that time) and measurement was performed in 2 repetitions for each sample. The hygroscopicity was expressed as the mass of adsorbed water per 100 g of the sample [17].

#### 2.3.4. Solubility

In brief, 0.5 g of the sample was dissolved in 50 mL of distilled water and then shaken for 30 min using a Multi Rotator RS-24 (BIOSAN, Otwock, Poland). The resulting mixture was then centrifuged using centrifuged MPW-352R (MPW, Warsaw, Poland) at 5000× *g* rpm. The supernatants were subsequently oven-dried at 105 °C until reaching a constant weight. The solubility of a sample was calculated as the percentage of the dried weight of the soluble to the initial weight [18].

#### 2.3.5. Color Measurement

The color measurement was performed using a portable calorimeter, NH310 (EnviSense, Lublin, Poland), according to the procedure proposed by Haas et al. [19]. Color coordinates L*, a*, and b* are described, respectively: brightness on a scale from 0 (black) to +100 (white), and the balance between red (+) and green (−) and between yellow (+) and blue (−). The total color difference (Δ*E*) was calculated as the root of the sum of the squares of the color coordinates examined. The value of Δ*E* between 3 and 5 is considered noticeable by the average observer.

#### 2.3.6. The Particle Size Distribution (PSD)

The particle size distribution (PSD) of powders was measured using a laser particle size analyzer (Malvern Mastersizer 3000, Malvern Instruments Ltd., Worcestershire, UK) with laser light scattering from 0.01 mm to 3.0 mm. In total, 5 g of the sample was prepared for the treatment. The PSD survey was conducted automatically through laser diffraction and based on the dry dispersion method. The particle size distribution was presented as d_10_, d_50_ (median diameter), and d_90_, which was referred to as the respective 10th, 50th, and 90th percentile of the total volume (assuming the spherical shape of the particles in the study). According to the formula provided by Dziki et al. [20], the volume-based size distribution (span) was calculated as

(2) $$span=\frac{d_{90}-d_{10}}{d_{50}}$$

#### 2.3.7. Morphology Properties

The powder microstructure was observed with a scanning electron microscope (SEM), VEGA LMU (TESCAN, Brno, Czech Republic), operating at an acceleration voltage of 10 kV. Before the research, prior to the analysis, the powders were frozen in liquid nitrogen and then lyophilized. A single sample of powder was placed on a carbon disc using special silver tape and then coated with gold using a vacuum sublimation, K-550 × (Emitech, Ashford, UK). Images were taken at 500× and 2000× magnification.

### 2.4. Biochemical Properties of Powders

#### 2.4.1. Water Extraction

In total, 0.4 µg of powder was extracted using 5 mL of distilled water. The extracts were initially shaken for 30 min using a Multi RS-60 rotator (Biosan, Otwock, Poland) and then centrifuged using an MPW-352R centrifuge (MPW, Warsaw, Poland) at a temperature of 4 °C for 10 min at 5000× *g* rpm. The extraction process was repeated and the resulting supernatant was collected for each sample and stored at −20 °C.

#### 2.4.2. In Vitro Digestion

The simulated digestion consisted of three phases, oral, gastric, and intestinal, and was based on the model proposed by Minekus et al. [21] with small changes. In total, 0.4 µg of the powder was given an oral digest where samples were treated with 0.8 mL of simulated salivary fluid (SSF), 0.005 mL of CaCl_2_, 0.695 mL of distilled water, and 0.1 mL of amylase (with an activity of a 75 U/mL digestive mixture), respectively. The pH in this phase was 7 (adjusted with 1 M NaOH or HCl as needed). In the oral phase, the samples were incubated in the dark for 2 min at 37 °C. Next, the oral phase was mixed with the gastric phase in a 1:1 ratio, through added 1.6 mL of simulated intestinal fluid SGF, 0.001 of CaCl_2_, 0.199 of distilled water, 0.1 mL of 6 M HCl, and 0.1 mL of pepsin (a 2000 U/mL digestive mixture), respectively, and the final pH was 3. In the gastric phase, the samples were incubated in the dark for 2 h at 37 °C. In the intestinal phase after passing through the oral and gastric phase trial, the following were added: 1.7 mL of simulated intestinal fluid (SIF), 0.008 mL of CaCl_2_, 0.059 mL of distilled water, 0.5 mL of 1 M NaOH, 1 mL of pancreatin (a 100 U/mL digestive mixture), and 0.5 mL of 10 mM bile salts, and the final pH was 7. In the intestinal phase, the samples were incubated in the dark for 2 h at 37 °C. After simulated digestion, the obtained extracts were centrifuged with an MPW-352R centrifuge (MPW, Warsaw, Poland) at a temperature of 4 °C for 10 min at 5000× *g* rpm. The extraction process was carried out twice and the resulting supernatant was collected for each sample and stored at −20 °C.

#### 2.4.3. Total Flavonoid Content (TFC)

TFC was determined using the methodology proposed by Nguyen et al. [22] with minor changes. A total of 20 µL of the extract was mixed with 180 µL of H_2_O and 10 µL of 5% NaNO_2_ and after 5 min with 10 µL of 10% AlCl_3_. After another 6 min, 40 µL of 1 M NaOH was added. Finally, after 20 min, the absorbance of the mixture was measured at a wavelength of 510 nm. TFC of evaluated samples was expressed as quercitin equivalents in mg per 100 g of powder solids.

#### 2.4.4. Total Anthocyanin Content (TAC)

Two separate dilutions were created using 20 µL of the extract: the first was mixed up with 80 µL of a 0.025 M KCl buffer at pH 1, and the second with 80 µL of a 0.04 M sodium acetate buffer at pH 4.5. After waiting for 15 min, the absorbance at wavelengths at 520 nm and 700 nm was measured for both solutions. Absorbance and total anthocyanins were calculated according to the formula proposed by Kopjar et al. [23]. TAC of evaluated samples was expressed as cyanidin 3-O-glucoside equivalents in mg per 100 g of powder solids. The assay was performed in 3 replicates.

#### 2.4.5. ABTS Radical Scavenging (AA)

In total, 10 µL of the extract (or solvent in the case of the control sample) was mixed with 250 µL of 7 mM ABTS with an initial absorbance of about 0.700. After 10 min, absorbance of the mixture was measured at a wavelength of 732 nm. The antioxidant activity (AA) of evaluated samples was expressed as Trolox equivalents in mg per g of powder solids. The assay was performed in 3 replicates [24].

#### 2.4.6. Ferric-Reducing Antioxidant Power (FRAP)

At the beginning, 50 µL of a 200 mM (pH 6.6) phosphate buffer, 50 µL of 1% potassium ferrocyanide, and 50 µL of the extract were added to each well of a 96-well plate. The mixture was then induced for 20 min at 50 °C. Then, 50 µL of 10% TCA was added thereto. In total, 100 µL of the previously prepared reaction mixture, 100 µL of distilled water, and 20 µL of 0.1% FeCl_3_ were taken into the next 96-well plate, respectively. The absorbance of the mixture was measured at the wavelength 732 nm; FRAP of evaluated samples was expressed as Trolox equivalents in mg per g of powder solids. The assay was performed in triplicate [25].

#### 2.4.7. Inhibition of the XO

In total, 20 µL of a 0.1 unit/mL xanthine oxidase (XO) solution, 140 µL of a 0.06 M phosphate buffer at pH 7.5, and 20 µL of the inhibitor (test extract) were poured into the well of the UV plate. The mixture thus formed and was incubated at 30 °C for 10 min. Then, 120 µL of 0.015 mM xanthine (substrate) was added and incubated again for 10 min at 30 °C. The absorbance was then measured at 295 nm. The control sample was performed in the same way, omitting the addition of the extract [26]. All measurements were performed in triplicate. One unit of inhibitor activity (IU) was defined as the activity that inhibits one unit of enzyme activity. The results were expressed in IU per g of d.s.

#### 2.4.8. Inhibition of the LOX

The initial reaction mixture contained 10 µL of a lipoxygenase (LOX) enzyme solution (167 U/mL), 240 µL of a 0.06 M phosphate buffer, and 10 µL of the inhibitor (test extract). The mixture was thus formed and was incubated for 5 min at 30 °C. Afterward, 40 µL of 2.5 mmol/L linoleic acid was added, incubated again under identical conditions before measuring the absorbance at 234 nm [27]. All measurements were performed in triplicate. One unit of LOX activity was defined as the activity oxidizing 0.12 µmole of linoleic acid per 1 min under reaction conditions. One unit of inhibitor activity (IU) was defined as the activity inhibiting one unit of enzyme activity. The results were expressed in kIU per g of d.s.

#### 2.4.9. Inhibition of the Activity of COX-2

The effect of the water and in vitro extracts on COX-2 (cyclooxygenase-2) activities was tested using a COX Colorimetric Inhibitor Screening Assay Kit (Cayman, No. 701050). All measurements were performed in triplicate. One unit of inhibitor activity (IU) was defined as the activity inhibiting one unit of enzyme activity. The results were expressed in kIU per g of d.s.

#### 2.4.10. Bioaccessibility

In vitro bioacceptability was calculated as the percentage ratio of the total content of bioactive components and the antioxidant or anti-inflammatory activity in the post-digested samples relative to the pre-digested samples [28].

### 2.5. Statistical Analysis of the Obtained Results

All experiments were conducted in repetitions unless otherwise stated. To compare means, a one-way analysis of variance (ANOVA) and a Tukey post hoc test were used. Moreover, the correlation coefficients between the variables were determined. All tests were performed at a significance level of α = 0.05 using Statistica 10.0 software (StatSoft, Inc., Tulsa, OK, USA).

## 3. Results and Discussion

### 3.1. Yield of Spray Drying and Basic Physical Properties of Powders

Table 2 shows the results of the measurement process yield (PY) for spray drying of the feed solution MAF, inulin, and pectin, as well as moisture content (MC), water activity (WA), hygroscopicity (H), and solubility (S) of obtained powders. PY ranged from 61.57% (M1) to 50.51% (M5). It was observed that the addition of pectin in the carrier solution above 2% had a significant effect on PY in comparison to the sample with only inulin in the recipe of wall material. In the literature, it can be found that a successful course of drying is considered when the powder recovery is above 50% [29], which was achieved in these studies. The decrease in drying efficiency could be caused by the use of a drying temperature above the glass transition temperature of the carrier materials, which ultimately resulted in the transformation of the carriers into the state of viscoelastic rubber and manifested itself sticking in the chamber [30]—especially since most of the commercially available inulin has a glass transition temperature oscillating between 120 and 125 °C [31] and in the case of pectin, it is 35 °C [32]. Additionally, our research observed a very strong negative and significant correlation between PY and MC (r = −0.89, *p* = 0.044). This is consistent with the observations of Cynthia and coworkers [33] that low moisture content causes it to limit the ability of water to act as a plasticizer, resulting in higher powder recovery. Researchers using inulin or pectin individually as carriers for curcumin and turmeric extracts reported PY levels ranging from 30.42% to 38.05% (pectin variants) and between 36.04 and 38.96% (inulin variants), respectively. During these tests, the carrier solids’ concentration in each case was only 1% [12], which may explain the higher PY in our studies—especially since there are several reports that increasing the carrier concentration in the solution results in a higher PY value [33,34].

In the case of MC (Table 2), it was observed that with the increase in the content of pectin in the powder, the moisture content also increased. Simultaneously, for both MC and WA, it was noted that the minimum addition of pectin, which replaces inulin, significantly affected the value of both obtained parameters. The MC value of the powders did not exceed 5%, which, according to Tontul and Topuz [29], serves as the basis for recognizing them as safe in terms of microbiology and allows for long-term storage. This is additionally supported by the fact that the WA was below 0.3, which not only limits the microbial growth, but also protects the food against the potential lipid oxidation, and Maillard reaction [35]. Furthermore, a positive correlation was also observed between MC and H (r = 0.78, *p* = 0.122), but it was not linearly significant. H ranged from 11.83 g/100 g for sample M1 to 12.46 g/100 g for M5; however, no statistically significant effect of variable carrier content on the parameter was observed. It is assumed that powders with a hygroscopicity below 20% are considered weakly hygroscopic [36]. In general, it was observed that the addition of pectin reduced the solubility of powders compared to the sample in which only the combination of MAF and inulin extracts was present. Solubility ranged from 84.85% (M3) to 91.29% (M1). Solubility depends on the size of the particles present in the powder. Smaller particles dissolve better than larger ones [37]. In our case, a negative correlation was observed between S and particle size in the 10th (r = −0.85, *p* = 0.66) and 90th (r = −0.81, *p* = 0.094) percentiles, but neither correlation was statistically significant.

### 3.2. Color Profile of Powders Based on an Extract of MAF

In the case of brightness (L*), this parameter is influenced by both the type of carrier and its concentration in the feed solution [38]. The value of the L* ranged from 83.17 (M1) to 84.69 (M4). When it comes to powders containing pectin in the composition, no significant differences in the measurement of this parameter were observed (Table 3). L* was very strong and positively correlated with MC (r = 0.87, *p* = 0.057), H (r = 0.85, *p* = 0.068), and a* (r = 0.82, *p* = 0.086), and negatively with S (r = −0.83, *p* = 0.078), but all correlations were not linearly significant. Regarding the a* coordinate (Table 3), the increasing amount of pectin caused powders to be perceived as more red. Simultaneously, a positive and significant correlation was observed between the a* coordinate and the total content of anthocyanins in powders (r = 0.93, *p* = 0.038), which suggests that the increase in pectin addition had a protective effect on bioactive compounds during spray drying. A similar conclusion was reached by Barańska et al. [39] during the spray drying of a sour cherry juice concentrate, where they found that a higher value of the a* is associated with better protection of anthocyanins through the carrier. The powders’ blueness (b*) was the highest for M5. In general, in the case of the parameter a* and b*, there was a significant variation among the tested samples. However, the total color difference (ΔE) indicated that the average observer would have difficulty distinguishing the powders.

### 3.3. The Particle Morphology of Powders Based on an Extract of MAF

**Figure 1:**
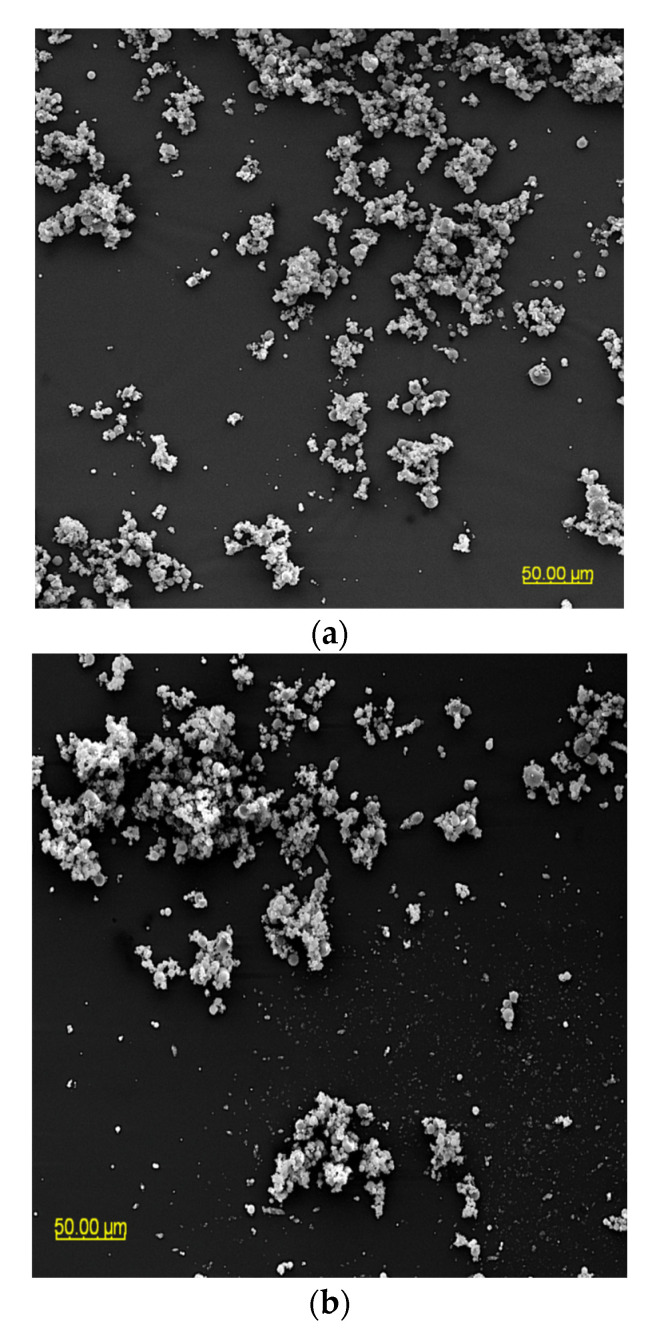

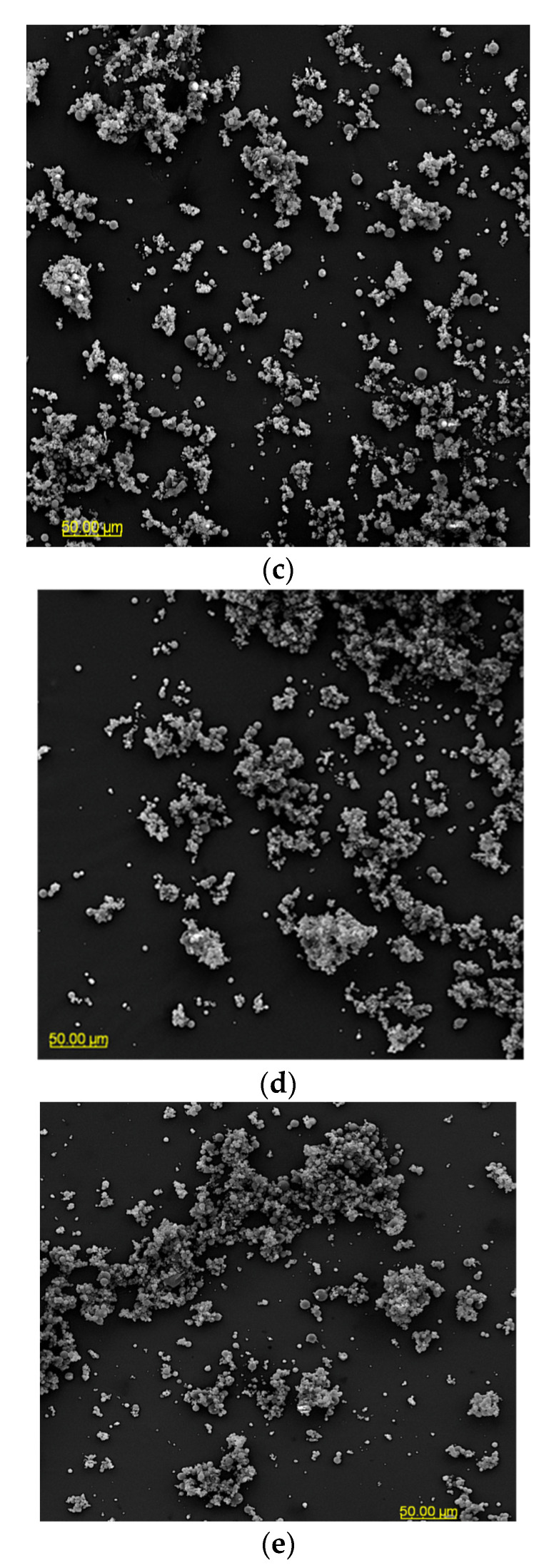
SEM microphotographs of powders based on an extract of *Malvae arboreae flos* (500× magnification): (**a**) M1—100% inulin in wall material, (**b**) M2—98% inulin in wall material and 2% pectin in wall material, (**c**) M3—96% inulin in wall material and 4% pectin in wall material, (**d**) M4—94% inulin in wall material and 6% pectin in wall material, and (**e**) M5—92% inulin in wall material and 8% pectin in wall material.

**Figure 2:**
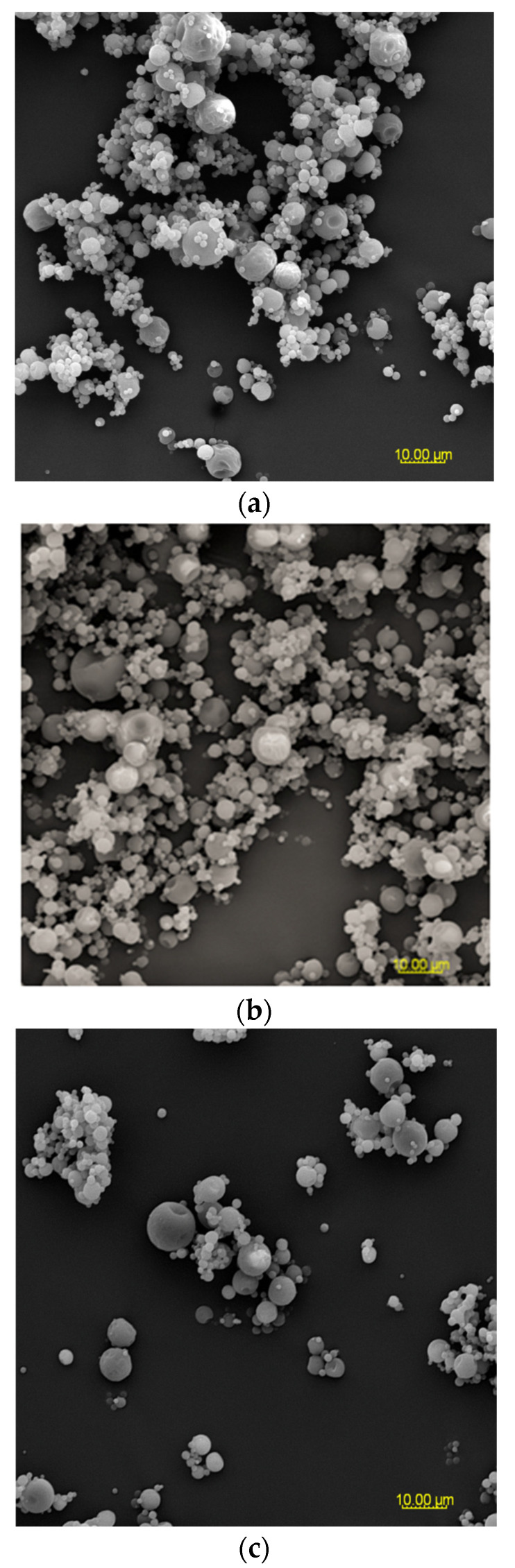

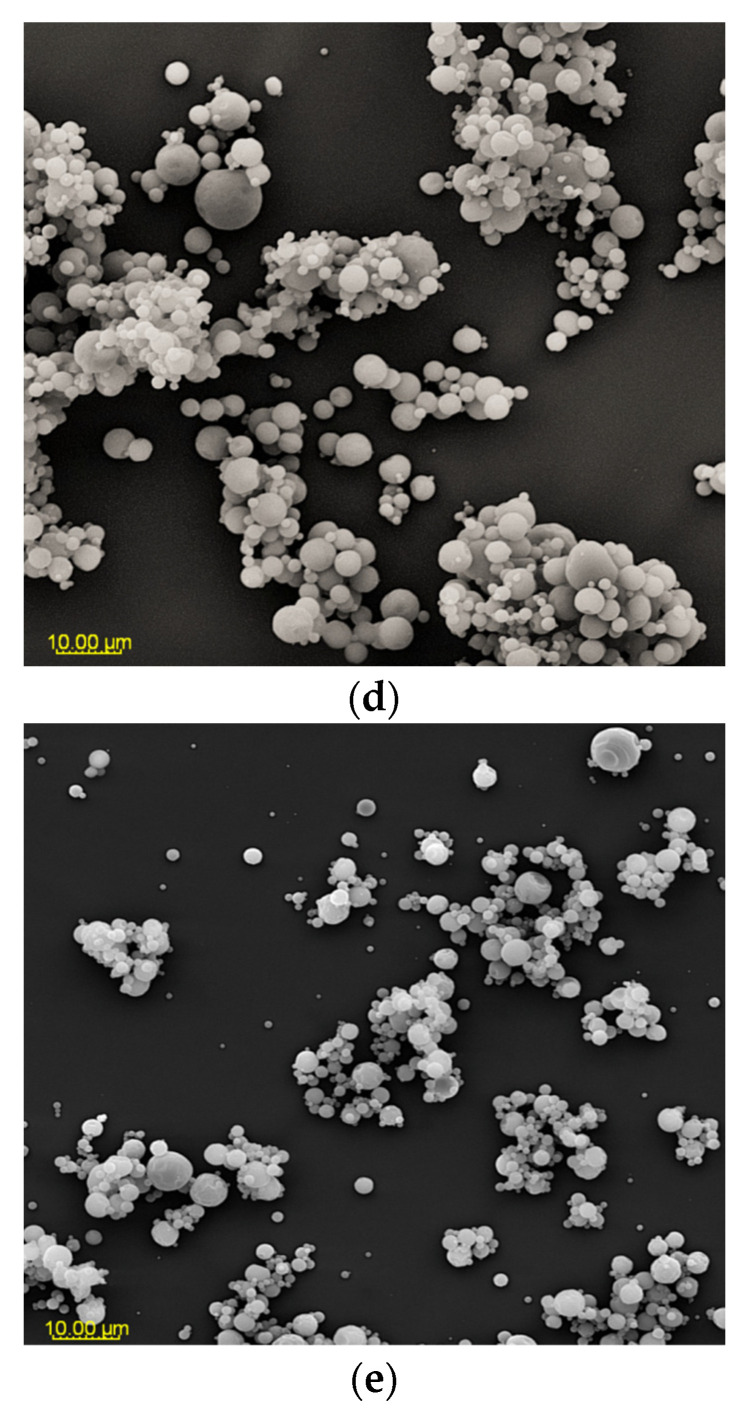
SEM microphotographs of powders based on an extract of *Malvae arboreae flos* (2000× magnification): (**a**) M1—100% inulin in wall material, (**b**) M2—98% inulin in wall material and 2% pectin in wall material, (**c**) M3—96% inulin in wall material and 4% pectin in wall material, (**d**) M4—94% inulin in wall material and 6% pectin in wall material, and (**e**) M5—92% inulin in wall material and 8% pectin in wall material.

The particle morphology of powders based on an extract of *Malvae arboreae flos* is shown in Figure 1 and Figure 2. When magnified at 500× (Figure 1), it was observed that the powder particles are clustered into agglomerates, with the most clusters being formed for the M1 and M2 samples. In contrast, more dispersion of particles is visible for the M3–M5 samples.

At 2000× magnification, it is evident that the shape of the sample particles is predominantly circular with a smooth surface. However, in the case of M1 and M2 samples, concave distortions are noticeable, especially in larger particles, along with cracks (Figure 2). Surfaces with open pores or cracks are associated with poorer protective properties, as they promote the loss of the core material [8]. Goëlo et al. [12], who employed inulin as an independent carrier, also observed spherical particles, but with a slightly rough structure. Researchers who combined maltodextrin with pectin (in the ratio of 74 g:6 g) in a 40% solution of the carrier reported that the particles had a smooth structure, and were small, round, and concave [11]. Sansone et al. [8], who mixed maltodextrin with pectin (a 10:1 ratio), also observed a similar morphology. The final conclusions from this research are that pectin serves as a coating agent, while maltodextrins primarily act as a matrix-forming material, resulting in better protection for polyphenols than using maltodextrin alone as a wall material. When combining pectin/sodium alginate (the weight ratio 1.5 g:1.5 g) as a 3% solution of carriers, it was noticed that at the temperature of 100 °C and 130 °C, the particles exhibited a spherical and smooth surface, whereas at the temperature of 160 °C and 190 °C, wrinkles and cracks appeared on their surface [40].

### 3.4. Particle Size Composition of Powders Based on an Extract of MAF

Determining the particle size composition is an important parameter because it provides useful information for the transport and storage because of particles. Moreover, the particle size is related to many physical parameters of the powder [41]. Additionally, the stability of bioactive compounds, which are sensitive to the environmental conditions, is also influenced by particle size [42]. The particle size distribution of powders based on an extract of *Malvae arboreae flos* is shown in Table 4. As for d50 and d90, the highest values were recorded for M3 (3.71 µm and 56.63 µm, respectively), while the lowest values were observed for M1 (3.13 µm and 7.73 µm, respectively). Additionally, strong negative correlations were also observed between d10, d90, and S (r = −0.85, *p* = 0.066 and r = −0.81, *p* = 0.094, respectively), although neither correlation was linearly significant. The volume-based size distribution (span) was highest for M3 (14.51), and the lowest for M2 (2.30). It was also noted that span exhibited a strong negative correlation with the solubility of the powders (r = −0.88, *p* = 0.092), but this correlation was not linearly significant. Pieczykolan et al. [11] observed that the differentiation in particle sizes, along with a high value of the span index, indicates that the samples have a lower tendency to form agglomerates, leading to uneven dissolution and pigment release [11]. These observations align with our findings.

### 3.5. Total Flavonoid and Anthocyanin Content of Powders Based on an Extract of MAF

The total content of bioactive compound powders based on an extract of MAF is presented in Table 5. In the case of total flavonoid content (TFC), it was observed that both before and after digestion, with the increase in the pectin content in the powder, the TFC also increased. Specifically, concerning the total flavonoids before digestion (TFC-BD), it was noticed that the addition of 4% pectin had a significant impact on the amount of the bioactive component. In the case of total flavonoid content after digestion (TFC-AD), it was observed that the addition of 2% pectin significantly affected the TFC value compared to the M1 sample. Total anthocyanin content before digestion (TAC-BD) and total anthocyanin content after digestion (TAC-AD) were generally higher where pectin and inulin were blended compared to samples containing only inulin. Regarding TAC-BD, the value differed statistically when the pectin addition was at least 4%. In the case of TAC-AD, even the minimum addition of pectin influenced the content of bioactive components. Similarly, Tarone et al. [43], using a mixture of pectin and inulin as an encapsulating agent for polyphenols recovered from a jabuticaba peel, observed higher values of total polyphenol content for mixed carriers than when using them individually. The existing literature also highlights that the combination of both inulin and pectin leads to the formation of highly branched structures, creating more space for the formation of hydrogen bonds, hydrophobic interactions, and electrostatic interactions [43]. This phenomenon helps explain the relationship between polyphenols and fiber fractions [44] and the increased TFC and TAC. In another study, it was noted that the pectin supplement in combination with a soy protein isolate exhibits superior anthocyanin release behavior and antioxidant stability compared to those produced using only a soy protein isolate [45]. In our studies, there was a decrease in both TFC and TAC after digestion compared to TFC-BD and TAC-BD (Table 5). Bioacceptability for the TFC ranged from 48.92% (M4) to 69.85% (M3) while for M1, it was 53.78%. However, the bioacceptability of the sample only with inulin as a carrier material significantly differed only in comparison to M3. In the case of the M3 sample, we observed a significant dispersion of molecules (Table 4, Figure 2c), resulting in uneven pigment release. Bioacceptability for the TAC was from 17.26% (M1) to 59.71% (M5). It was observed that replacing inulin with 6% or more of pectin had a significant effect on bioacceptability compared to the sample without pectin in the recipe. Regarding flavonoids, their decline after digestion can be explained by interactions with digestive enzymes [46]. In the case of quercetin, which is also present in the hollyhock flower, it can be assumed that its decrease (in relation to TFC) was due to interactions occurring at the salivary level, as it has been demonstrated that the flavonoid inhibits α-amylase [47]. The increase in α-amylase inhibitory activity is affected by the planarity of flavonoids associated with the lack of saturation of C2–C3 bonds [46]. Furthermore, Zeng et al. [48] tested 10 flavonoids, including quercetin and kaempferol, and observed that these compounds also interact with pepsin, reducing its activity. These tested compounds effectively suppressed pepsin fluorescence through static quenching and spontaneously bonded to pepsin, primarily through electrostatic forces, hydrophobic interactions, and hydrogen bonding. The inhibition of enzyme activity occurred as a result of changing the microenvironment of the active center of pepsin using flavonoids [48]. The combination of flavonoids with proteins, including enzymes, leads to the loss of their properties, especially antioxidant properties. When attempting to enhance the oral bioavailability of flavonoids, a high-protein diet may diminish these properties [46]. Therefore, it may be appropriate to consider encapsulating flavonoids in microcapsules to limit their contact with enzymes. Additionally, it should be noted that in the intestinal section, a pancreatin enzyme is used. Here, too, enzyme activity may be inhibited using flavonoids. Gatto et al. [49], while studying quercetin (also present in MAF), observed that the increase in C3 position hydroxyl moieties affected the inhibitory activity of the flavonoid, indicating that changes in the position of the flavonoid skeletons translated into the inhibitory effect of lipases. Furthermore, the decrease in anthocyanin content after digestion can be explained by their sensitivity to the pH of the environment, particularly their instability under alkaline conditions. This instability is associated with structural changes of the flavylium cation to a colorless, less stable chalcone [50]. Barańska et al. [39] also noted a decrease in the total amount of anthocyanins after digestion in samples that were spray-dried powders with carriers, specifically maltodextrin/pea protein based on a sour cherry juice concentrate. A decrease in the content of cyanidin 3-O-glucoside in powders after digestion was also observed when the carrier was maltodextrin and the core anthocyanins derived from a purple potato extract [51]. Many studies focus on the consumption of foods rich in anthocyanins; however, they consistently conclude that the total anthocyanin content remains low in its unchanged form in urine or blood [52]. Therefore, attempts to protect them during digestion by using spray drying with the addition of a carrier are considered beneficial.

### 3.6. Antioxidant Properties of Powders Based on an Extract of MAF

The free radical scavenging capacity of ABTS (AA) and ferric-reducing antioxidant power (FRAP) of powders based on MAF aqueous extracts are presented in Table 5. AA content of the powders derived from MAF ranged from 10.15 mg Trolox/g solids to 12.63 mg Trolox/g solids before digestion and from 4.17 mg Trolox/g solids to 11.00 mg Trolox/g solids after digestion. A similar ability to scavenge free radicals against ABTS (10.28, 12.34, and 12.60 mg Trolox/g solids) was observed after drying the superfood black garlic with 10% maltodextrin. However, in those studies, the process was conducted at a temperature of 50 °C or 70 °C and lasted for 18 or 30 h [53], whereas in our study, the drying of the entire prepared sample took no longer than 30 min. Regarding the FRAP values of the powders (Table 5), they ranged from 2.70 mg Trolox/g solids to 3.18 mg Trolox/g solids before digestion and from 4.32 mg Trolox/g solids to 5.01 mg Trolox/g solids after digestion. Authors who used similar carriers for encapsulating polyphenols with a jabuticaba peel observed a significantly lower FRAP value for their powders at the level of 343.1 μmol Trolox/100 g solids (0.86 mg Trolox/g solids) [43]. This indicates that *Malvae arboreae flos*, and at the same time, edible flowers, are valuable alternative antioxidants, especially considering that only 0.5 g of MAF was used in each tested case during our study.

The antioxidant activity of powders before digestion (-BD) showed no significant difference for AA and FRAP. After digestion (-AD), significant changes in AA values were observed in all pectin-containing samples compared to the M1 sample—similar in most cases for FRAP. Regarding AA-AD, a positive correlation was observed between the total content of flavonoids (r = 0.87, *p* = 0.057, and r = 0.87, *p* = 0.058) and anthocyanins (r = 0.89, *p* = 0.042, and r = 0.78, *p* = 0.121) in powders before and after digestion, respectively. However, the only significant correlation was found between AA-AD and TAC-AD, indicating that the linear increase in anthocyanin content primarily contributes to their ability to scavenge the ABTS free radical. Additionally, when examining anthocyanins derived from a red radish, a strong correlation was also observed between their content and post-digestion antioxidant activities [54]. Bioaccessibility for AA was higher in samples containing pectin in the composition. This result may stem from the interaction between the inulin–pectin carrier mixture and the bioactive compounds described in the preceding section. On the other hand, in the case of FRAP, the sample containing only inulin as a carrier exhibited higher bioaccessibility compared to the others, though in all cases, bioacceptability exceeded 100%. A similar increase in FRAP-AD in relation to FRAP-BD (resulting in bioacceptability exceeding 100%) was observed during the spray drying of a cherry juice concentrate, where maltodextrin and pea protein were selected as carriers [39].

In the case of spray drying of the polyphenol extract from the jabuticaba peel, where the carrier was inulin with pectin, higher FRAP values were observed compared to powders containing only pectin or pectin with trehalose as wall material [43]. Concerning FRAP-AD, it was noticed that the reduced flavonoid content after digestion (r = −0.82, *p* = 0.089) influenced a higher capacity for reducing iron ions. This suggests that flavonoids associated with pectin form complexes with poorer reducing properties, although this association was not statistically significant. This is further supported by a positive correlation between FRAP-AD and S (r = 0.77, *p* = 0.124), but it is not linearly significant. The addition of pectins reduced the solubility of powders, leading to a decrease in the amount of released active substances.

### 3.7. Anti-Inflammatory Properties of Powders Based on an Extract of MAF

The inhibitor activity (IU) was higher for all powders containing pectin in the recipe before digestion (-BD) compared to the sample without pectin, for the xanthine oxidase (XO), lipoxygenase (LOX), and cyclooxygenase-2 (COX-2) inhibition analysis (Table 5). However, after digestion (-AD), the samples did not exhibit a differential ability to inhibit xanthine oxidase but significant differences were observed in the inhibition of lipoxygenase, particularly for M1. In the case of the inhibition of cyclooxygenase-2 after digestion, the ability of the powders to inhibit the enzyme increased with the pectin content. The bioaccessibility of anti-inflammatory activity in the post-digested samples relative to the pre-digested samples of powders was highest in the case of the M1 sample for the XO, LOX, and COX-2 analysis. However, it decreased with the increase in the pectin content in the case of XO, LOX, and COX-2 (except for M5). In addition, a positive correlation was observed between XO-AD and the amount of anthocyanins in the powders before digestion (r = 0.68, *p* = 0.206) and AA-AD (r = 0.71, *p* = 0.182), but these were statistically insignificant correlations. Yang et al. [55] also confirmed that anthocyanins exhibited efficient antioxidant capacity and inhibitory activity on XO as a digestion product. Furthermore, in their quest for xanthine oxidase inhibitors, researchers have identified nine flavonoids within the *Phyllanthus* genus (quercetin, kaempferol, rutin, apigenin, luteolin, myricetin, catechin, epicatechin, and epigallocatechin), all of which demonstrate inhibitory activity against the tested enzyme [56]. Importantly, it has been established that quercetin and kaempferol are present in *Malvae arboreae flos*. The search for XO inhibitors is important because excessive activity of the enzyme can lead to an increase in oxidative stress [57], cardiovascular diseases [58], or the development of Type 2 Diabetes [59]. Złotek et al. [60] noted that products in an in vitro model of digestion may inhibit the action of lipoxygenase, which is desirable for health. In our studies, the most effective inhibitor LOX was found in samples M1 and M2 after digestion (Table 5). Researchers studying bread with the added dried Saskatoon berry also observed an increased ability to inhibit LOX in the post-digestion product compared to pre-digestion, indicating that such products can help prevent the formation of reactive oxygen species in our body. However, the values obtained in this study were significantly higher than those obtained when testing MAF powders [61]. In our research, inhibition of COX-2-AD was observed to be positively correlated with the total content of flavonoids (r= 0.95, *p* = 0.011 and r = 0.75, *p* = 0.104) and anthocyanins (r = 0.83, *p* = 0.081 and r = 0.96, *p* = 0.008) before and after digestion, respectively. However, only the correlation between COX-2-AD and TFC-BD and TAC-AD was statistically significant. Lachowicz et al. [61] also found a link between the total polyphenol content of the sample and its ability to inhibit COX-2. The researchers used maltodextrin and inulin encapsulated with the Saskatoon berry as the core. Additionally, wheat bread was fortified with the obtained powders, and it was noted that these bread samples exhibited a much higher COX-2 inhibition capacity than fruit powders without the added wall material after being incorporated into the bread [61]. This demonstrates the viability of producing powders with carbohydrate carriers as a potential source of bioactive compounds, aligning with our study.

## 4. Conclusions

The present research demonstrates the possibility of producing microcapsules based on a *Malvae arboreae flos* edible flower extract, using inulin and pectin as coating agents in a varying ratio from 98:2 to 92:8, which ensures efficient powder recovery. The resulting powders exhibited low moisture content and water activity. Increasing the proportion of pectin, replacing inulin, resulted in a higher hygroscopicity and a reduced solubility in water. Furthermore, there was also no significant difference in the total color difference between the powders. Additionally, it was observed that the increase in the pectin content reduced the tendency of the powders to form agglomerates, while also reducing the number of cracks and distortions among the tested particles. The increase in the content of pectins in the wall of microcapsules had a protective effect on the total content of flavonoids and anthocyanins; however, their bioacceptability varied. The powders demonstrated a significant increase in ferric-reducing antioxidant ability and inhibition relative to xanthine oxidase and cyclooxygenase-2 after in vitro digestion. These findings suggest that the tested powders could serve as a promising source of compounds with antioxidant and anti-inflammatory properties. It is important to note, however, that these studies were conducted using an in vitro model and future in vivo studies will be necessary to draw full conclusions.

## Figures and Tables

**Table 1:** Biochemical properties of aqueous extracts prepared on the basis of MAF.

Parameter	Value
TFC (mg quercitin/g solids)	122.97 ± 5.74
TAC (mg cyanidin 3-O-glucoside/g solids)	7.63 ± 0.45
AA (mg Trolox/g solids)	175.22 ± 5.98
FRAP (mg Trolox/g solids)	79.89 ± 0.38
XO (IU/g solids)	1.69 ± 0.01
LOX (kIU/g solids)	0.39 ± 0.04
COX-2 (kIU/g solids)	3100.78 ± 116.55

TFC—total flavonoid content; TAC—total anthocyanin content; AA—ABTS radical scavenging; FRAP—ferric-reducing antioxidant power; XO—inhibition of the xanthine oxidase; LOX—inhibition of the lipoxygenase; COX-2—inhibition of the cyclooxygenase-2.

**Table 2:** Process yield (PY) of spray drying, moisture content (MC), water activity (WA), hygroscopicity (H), and solubility (S) of powders based on an extract of *Malvae arboreae flos*.

Sample	PY (%)	MC (%)	WA (−)	H (g/100 g)	S (%)
M1	61.57 ± 1.15 ^d^	2.45 ± 0.01 ^a^	0.229 ± 0.00 ^d^	11.83 ± 0.79 ^a^	91.29 ± 0.58 ^b^
M2	59.25 ± 1.23 ^cd^	3.25 ± 0.01 ^b^	0.209 ± 0.00 ^b^	12.22 ± 0.20 ^a^	87.94 ± 0.35 ^ab^
M3	55.03 ± 0.49 ^b^	3.32 ± 0.01 ^b^	0.216 ± 0.00 ^c^	12.12 ± 0.13 ^a^	84.85 ± 1.00 ^a^
M4	57.59 ± 0.53 ^bc^	3.81 ± 0.01 ^c^	0.201 ± 0.00 ^a^	12.45 ± 0.08 ^a^	85.96 ± 0.96 ^a^
M5	50.51 ± 0.59 ^a^	4.55 ± 0.02 ^d^	0.235 ± 0.00 ^e^	12.46 ± 0.25 ^a^	86.83 ± 1.08 ^ab^

M1—100% inulin in wall material, M2—98% inulin in wall material and 2% pectin in wall material, M3—96% inulin in wall material and 4% pectin in wall material, M4—94% inulin in wall material and 6% pectin in wall material, and M5—92% inulin in wall material and 8% pectin in wall material; ^a–e^—means indicated with different letters in columns are significantly different (α = 0.05).

**Table 3:** Color profile of powders based on an extract of *Malvae arboreae flos*.

Sample	L* (−)	a* (−)	b* (−)	∆E (−)
M1	83.70 ± 0.07 ^a^	2.70 ± 0.02 ^a^	−6.87 ± 0.01 ^d^	-
M2	84.43 ± 0.49 ^b^	3.30 ± 0.07 ^b^	−6.30 ± 0.08 ^a^	1.11
M3	84.40 ± 0.19 ^b^	4.09 ± 0.04 ^c^	−6.65 ± 0.03 ^b^	1.57
M4	84.69 ± 0.23 ^b^	4.50 ± 0.01 ^d^	−6.80 ± 0.05 ^d^	2.05
M5	84.64 ± 0.09 ^b^	5.37 ± 0.04 ^e^	−7.37 ± 0.03 ^c^	2.88

L*—brightness, a*—the balance between red (+) and green, b*—the balance between yellow (+) and blue, and ∆E—the total color difference; M1—100% inulin in wall material, M2—98% inulin in wall material and 2% pectin in wall material, M3—96% inulin in wall material and 4% pectin in wall material, M4—94% inulin in wall material and 6% pectin in wall material, and M5—92% inulin in wall material and 8% pectin in wall material; ^a–e^—means indicated with different letters in columns are significantly different (α = 0.05).

**Table 4:** The particle size distribution of powders based on an extract of *Malvae arboreae flos*.

Sample	d_10_ (µm)	d_50_ (µm)	d_90_ µm (−)	Span (−)
M1	0.52 ± 0.00 ^a^	3.34 ± 0.04 ^ab^	8.68 ± 0.13 ^a^	2.44 ± 0.01 ^a^
M2	0.53 ± 0.02 ^ab^	3.13 ± 0.11 ^a^	7.73 ± 0.27 ^a^	2.30 ± 0.04 ^a^
M3	0.64 ± 0.03 ^c^	3.71 ± 0.08 ^b^	56.63 ± 8.52 ^b^	14.51 ± 1.96 ^b^
M4	0.60 ± 0.03 ^bc^	3.65 ± 0.29 ^ab^	36.79 ± 19.38 ^ab^	9.58 ± 4.85 ^ab^
M5	0.55 ± 0.02 ^ab^	3.43 ± 0.15 ^ab^	18.45 ± 10.34 ^ab^	5.11 ± 2.73 ^a^

M1—100% inulin in wall material, M2—98% inulin in wall material and 2% pectin in wall material, M3—96% inulin in wall material and 4% pectin in wall material, M4—94% inulin in wall material and 6% pectin in wall material, and M5—92% inulin in wall material and 8% pectin in wall material; ^a–c^—means indicated with different letters in columns are significantly different (α = 0.05).

**Table 5:** The total content of bioactive compounds, antioxidant, anti-inflammatory and bioaccessibility (BA) in vitro in before-digestion (BD) and after-digestion (AD) powders based on an extract of *Malvae arboreae flos*.

Sample		M1	M2	M3	M4	M5
BD	TFC	280.43 ± 12.43 ^a^	284.09 ± 16.31 ^a^	325.55 ± 13.96 ^b^	407.24 ± 14.22 ^b^	419.43 ± 13.13 ^b^
	TAC	13.15 ± 1.71 ^a^	19.17 ± 1.23 ^ab^	20.32 ± 2.61 ^b^	19.52 ± 2.44 ^b^	23.48 ± 3.78 ^b^
	AA	11.89 ± 01.22 ^a^	10.15 ± 1.37 ^a^	11.32 ± 1.73 ^a^	12.63 ± 1.28 ^a^	11.14 ± 1.13 ^a^
	FRAP	2.70 ± 0.28 ^a^	2.99 ± 0.24 ^a^	3.18 ± 0.41 ^a^	3.07 ± 0.40 ^a^	2.85 ± 0.19 ^a^
	XO	0.17 ± 0.00 ^a^	0.20 ± 0.01 ^b^	0.24 ± 0.01 ^c^	0.26 ± 0.00 ^b^	0.26 ± 0.00 ^b^
	LOX	0.10 ± 0.01 ^a^	0.35 ± 0.00 ^b^	0.40 ± 0.00 ^c^	0.40 ± 0.00 ^c^	0.41 ± 0.00 ^d^
	COX-2	2.66 ± 0.14 ^a^	4.25 ± 0.58 ^a^	10.16 ± 1.34 ^b^	17.37 ± 0.87 ^c^	16.66 ± 2.61 ^c^
AD	TFC	150.38 ± 7.04 ^a^	178.83 ± 15.21 ^b^	205.24 ± 10.56 ^b^	199.15 ± 7.04 ^b^	205.24 ± 13.33 ^b^
	TAC	2.20 ± 0.30 ^a^	3.64 ± 0.43 ^b^	3.73 ± 0.33 ^b^	6.89 ± 0.72 ^c^	13.68 ± 0.32 ^d^
	AA	4.17 ± 0.20 ^a^	8.17 ± 0.49 ^b^	7.97 ± 0.63 ^b^	10.78 ± 1.18 ^c^	11.00 ± 1.30 ^c^
	FRAP	5.01 ± 0.07 ^b^	4.52 ± 0.18 ^a^	4.32 ± 0.29 ^a^	4.73 ± 0.20 ^ab^	4.35 ± 0.11 ^a^
	XO	0.30 ± 0.01 ^a^	0.31 ± 0.01 ^a^	0.31 ± 0.00 ^a^	0.31 ± 0.00 ^a^	0.31 ± 0.00 ^a^
	LOX	0.39 ± 0.01 ^d^	0.32 ± 0.01 ^c^	0.32 ± 0.03 ^bc^	0.31 ± 0.00 ^b^	0.23 ± 0.00 ^a^
	COX-2	7.02 ± 0.06 ^a^	10.20 ± 1.47 ^a^	11.77 ± 1.92 ^a^	17.65 ± 0.96 ^b^	23.15 ± 2.42 ^c^
BA (%)	TFC	53.78 ± 4.72 ^ab^	63.36 ± 8.40 ^bc^	69.85 ± 1.60 ^c^	48.92 ± 1.12 ^a^	48.96 ± 3.07 ^a^
	TAC	17.26 ± 4.69 ^a^	19.12 ± 3.08 ^a^	18.64 ± 2.55 ^a^	35.90 ± 5.76 ^b^	59.71 ± 9.04 ^c^
	AA	35.56 ± 5.15 ^a^	81.87 ± 11.62 ^bc^	71.32 ± 6.54 ^b^	86.15 ± 12.39 ^bc^	98.58 ± 3.23 ^c^
	FRAP	186.89 ± 17.68 ^b^	152.02 ± 9.27 ^a^	136.78 ± 8.79 ^a^	156.59 ± 18.33 ^ab^	153.17 ± 8.64 ^a^
	XO	173.1 ± 5.81 ^c^	155.5 ± 7.45 ^b^	128.6 ± 2.62 ^a^	120.9 ± 1.72 ^a^	120.4 ± 3.20 ^a^
	LOX	382.81 ± 26.46 ^c^	104.39 ± 1.66 ^b^	79.54 ± 7.93 ^ab^	77.73 ± 0.26 ^ab^	55.89 ± 0.91 ^a^
	COX-2	264.91 ± 14.76 ^c^	239.54 ± 7.18 ^c^	115.67 ± 10.49 ^ab^	102.03 ± 9.51 ^a^	140.09 ± 7.43 ^b^

TFC—total flavonoid content (mg quercitin/100 g solids); TAC—total anthocyanins content (mg cyanidin 3-O-glucoside /100 g solids), AA—ABTS radical-scavenging (mg Trolox/g solids); FRAP—ferric reducing antioxidant power (mg Trolox/g solids); XO—Inhibition of the xanthine oxidase (IU/g solids); LOX—Inhibition of the lipoxygenase (kIU/g solids); COX-2—Inhibition of the cyclooxygenase-2 (kIU/g solids); M1—100% inulin in wall material, M2—98% inulin in wall material and 2% pectin in wall material, M3—96% inulin in wall material and 4% pectin in wall material, M4—94 inulin in wall material and 6% pectin in wall material, M5—92% inulin in wall material and 8% pectin in wall material; ^a–d^—means indicated with different letters in rows are significantly different (α = 0.05).

## Data Availability

The data presented in this study are available on request.
